# Sensitive Drone Mapping of Methane Emissions without the Need for Supplementary Ground-Based Measurements

**DOI:** 10.1021/acsearthspacechem.1c00106

**Published:** 2021-07-28

**Authors:** Magnus Gålfalk, Sören Nilsson Påledal, David Bastviken

**Affiliations:** † Department of Thematic Studies—Environmental Change, Linköping University, 581 83 Linköping, Sweden; ‡ Tekniska verken i Linköping AB, Box 1500, 581 15 Linköping, Sweden

**Keywords:** climate change, methane, method development, drone, wastewater treatment

## Abstract

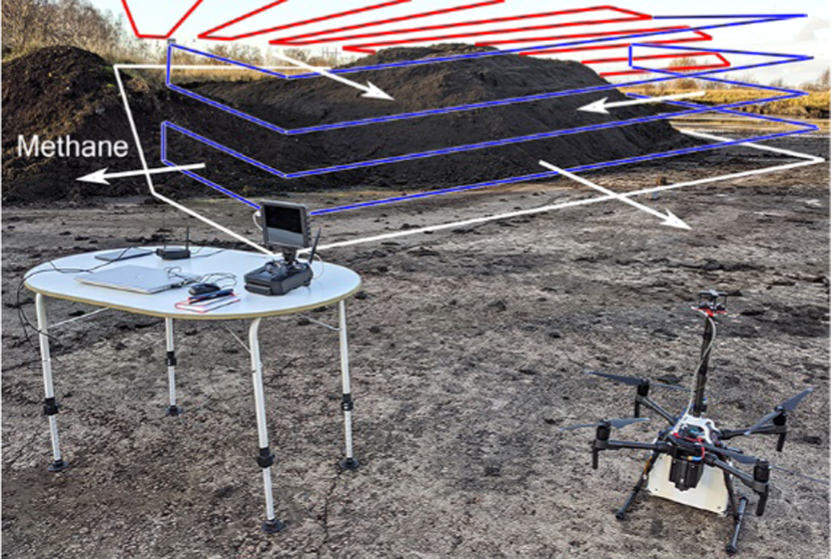

Methane (CH_4_) is one of the main greenhouse gas for which sources and sinks are poorly constrained and better capacity of mapping landscape emissions are broadly requested. A key challenge has been comprehensive, accurate, and sensitive emission measurements covering large areas at a resolution that allows separation of different types of local sources. We present a sensitive drone-based system for mapping CH_4_ hotspots, finding leaks from gas systems, and calculating total CH_4_ fluxes from anthropogenic environments such as wastewater treatment plants, landfills, energy production, biogas plants, and agriculture. All measurements are made on-board the drone, with no requirements for additional ground-based instruments. Horizontal flight patterns are used to map and find emission sources over large areas and vertical flight patterns for total CH_4_ fluxes using mass balance calculations. The small drone system (6.7 kg including batteries, sensors, loggers, and weather proofing) maps CH_4_ concentrations and wind speeds at 1 Hz with a precision of 0.84 ppb/s and 0.1 m/s, respectively. As a demonstration of the system and the mass balance method for a CH_4_ source that is difficult to assess with traditional methods, we have quantified fluxes from a sludge deposit at a wastewater treatment plant. Combining data from three 10 min flights, emission hotspots could be mapped and a total flux of 178.4 ± 8.1 kg CH_4_ d^–1^ was determined.

## 1 Introduction

Our ability to predict and mitigate climate change is highly dependent on the quantitative understanding of greenhouse gas (GHG) emissions and their regulation. The global decision to adopt climate goals of temperature change (rather than actual GHG emissions; COP21, Paris 2015) substantially increases our knowledge requirements because all GHG sources and sinks affect temperature. GHG emissions and their feedbacks have to be properly quantified to guide decisions toward the climate goals. Hence, there is a clear need to develop a better capacity to identify unknown sources and sinks on a local to landscape scale and to monitor that actions taken to reduce emissions have the desired effect. Methane (CH_4_) has 86 times higher global warming efficiency than carbon dioxide (CO_2_) per kg of gas on a 20 year time scale, is increasing at a high but irregular rate for partly unknown reasons, and is now at its highest level during the last 800 000 years.^1^ This implies that efforts to find and reduce CH_4_ emissions will be efficient for reducing global warming, but for this, we currently lack methods that are accurate, easy to use, and commonly available.

The European Green Deal^2^ has the aim of no GHG net emissions by 2050, with a high focus on reduction of CH_4_ emissions. The associated EU Methane strategy adopted in October 2020^3^ involves an increased ambition in CH_4_ monitoring in the energy, agriculture, and waste sectors, as these areas account for most of the anthropogenic CH_4_ emissions. For this, improved detection methods for hotspots and measurements of different fluxes are needed. Measurement techniques for such anthropogenic emissions should ideally be both sensitive (<a few hundred ppb), fast (1 Hz measurements), and have the ability to cover both small and large surface areas (>10 000 m^2^) at a high spatial resolution (a few m^2^) to identify hotspots and to estimate total emissions from a specific footprint area. Examples of application include monitoring large landfills for fugitive CH_4_ emissions and leak detection along gas pipelines (also being an economic incentive for companies handling natural gas and biogas).

Open and closed surface flux chambers represent a commonly used method approach for measuring anthropogenic surface emissions,^4−6^ having high accuracy and a well-defined but very small footprint, working well in smaller areas. Although large chambers can be used, measurements become impractical and very laborious for larger surfaces, especially in environments with heterogeneous emissions. It has the advantage of high sensitivity, capturing both uptake and emission. For a larger area, chambers often lead to underestimation of the total flux as emission hotspots are often not properly represented using a few measurement points.^4^ This can lead to a large total flux uncertainty, e.g., one study showed a fourfold underestimation compared to a site-wide flux measurement approach.^7^ Underpressurization or overpressurization of a chamber and disturbance of air motion within a chamber can also affect the flux from the contained area being measured,^8^ preventing the use of flux chambers in some types of environments.

On a larger scale, there are methods involving both passive infrared (IR) background radiation and active (laser) remote sensing, as well as micrometeorological approaches often based on Eddy covariance (EC). EC relies on the vertical mixing of emitted gas by turbulent eddies in the atmospheric inversion layer and a mast or tower with sensitive gas sensing and three-dimensional (3D) anemometers mounted at one or several heights, measuring the vertical air motion. Interpretation of flux tower data relies on the surrounding topology and assumptions of homogeneous surroundings and generates emission estimates of larger areas with a footprint that depends on wind direction and wind speed (thereby having a dynamic size and location). One application for EC is measuring GHGs from agricultural soils, but it has also been used on landfills for estimating CH_4_ fluxes.^9^

Passive thermal IR remote sensing of CH_4_ uses the difference in heat radiation between a background (such as the ground or a building) and the target gas to visualize and quantify fluxes through absorption and re-emission of light in the 3.3 μm (weaker) or 7.7 μm (stronger) CH_4_ bands. A commonly used method for leak detection is a handheld thermal camera for inspection, using a narrow-band spectral region (e.g., FLIR GF320, targeting the spectral range of 3.2–3.4 μm) that contains CH_4_ absorption features. As this method does not allow quantification of emissions and cannot separate between different gases that absorbs in this spectral region, it is mainly used to find possible leaks for follow-up measurements using other methods. For quantification of fluxes, spectroscopy is needed, which has been done both from the air at high altitudes^10−12^ and from the ground using a hyperspectral camera that can both identify and quantify emission sources in a landscape at a high spatial resolution.^13,14^

Active IR remote sensing techniques instead involve a laser-emitting light at wavelengths where CH_4_ absorbs and a reflector or backscattering by the atmosphere. By comparing the emitted and returned lights, the total absorption for a line of sight can be calculated, allowing mapping in several directions and across a vertical plane perpendicular to the wind direction. Differential absorption LiDAR uses a tunable laser to send pulsed light at two wavelengths: one that is easily absorbed by CH_4_ and another in a nearby continuum wavelength where CH_4_ does not absorb.^4^ A version of this technique is the open-path tunable diode laser absorption spectroscopy (TDLAS), which can be made light enough for an unmanned aerial vehicle (UAV) such as a quadcopter drone and used for vertical mapping of CH_4_ from above.^15^ A large uncertainty with downward-pointing instruments flying at a certain altitude, making column density scans, is that for flux calculations to be reliable, wind data is needed (speed and direction) for many different altitudes down to the ground. Lacking a wind profile can cause flux uncertainties as large as >70%.^15^ Plume modeling is another method that has been used for total area emission estimates at a large distance downwind from a source, either sampling CH_4_ concentrations directly and using inverse modeling or combined with simultaneous tracer gas releases relating CH_4_ emissions to another more easily measurable gas.^4^

On an even larger scale, large CH_4_ leaks have also been discovered using remote sensing from space, e.g., the TROPOMI instrument on-board the Sentinel-5P satellite launched in October 2017.^16^ A current limitation is the low sensitivity and spatial resolution (e.g., 7 × 7 km^2^ for TROPOMI). Airplanes have been used for mass balance calculations of total CH_4_ emissions, mapping a vertical plane downwind of a landfill.^17^

UAV-based mass balance calculation (fixed wing and rotary) is a new method for calculating total emissions on a small to large scale that has made fast progress in recent years due to development of low-weight sensors.^18^ Kunz et al.^19^ used a 1 kg nondispersive infrared CO_2_ sensor (SenseAir) on a UAV with an accuracy of 1.2 ppm to measure CO_2_ profiles. UAV-based mapping of CH_4_ in a horizontal plane at a constant altitude combined with inverse plume modeling has been used to estimate the total emission from a landfill^20^ using a low-weight (MQ-4 platform) semiconductor sensor working in the range of 10–10 000 ppm with an accuracy of 27 ppm.^21^ Andersen et al.^22^ developed a UAV-based AirCore system for sampling of GHGs in a 50 m long tube inside a UAV, analyzed minutes after landing, having a horizontal spatial resolution of 30 m. Golston et al.^23^ developed an open-path sensor using wavelength modulation spectroscopy in the 3.3 μm CH_4_ absorption band flown on a hexacopter with flight times of 5 min and a sensitivity of 10 ppb/s to monitor CH_4_ concentrations over time at different altitudes. Allen et al.^24,25^ introduced a downwind mass balance method to calculate the total CH_4_ flux from a landfill using proxy CH_4_/CO_2_ ratios from fixed wing CO_2_ mapping, combined with vertical profiles from a rotary UAV powered by a 100 m long tether, also including a Teflon tube to a stationary LGR-UGGA instrument on the ground. Wind measurements were made using a weather station on the ground. Schuyler et al.^26^ optimized the performance of a semiconducting sensor (MiCS-6814), designed for CH_4_ concentrations above 1000 ppm to be used at ambient levels of ∼2 ppm with an accuracy of 25 ppb and a precision of 180 ppb by calibrating the detector response to environmental parameters (temperature, relative humidity, and air pressure) and used this low-weight sensor on a DJI Phantom 3 to measure vertical CH_4_ profiles.

It was noted in both Allen et al.^25^ and Villa et al.^27^ that there were no fast high precision CH_4_ instruments (defined as better than 100 and 40 ppb/s at 1σ, respectively) available for small UAVs (<7 kg) on the market but that this was a possible future improvement and that simultaneous wind measurements ideally should be made using an instrument on the UAV. Shah et al.^28^ used two UAVs to measure a controlled CH_4_ release: one with an on-board anemometer and a ground-based near-IR CH_4_ analyzer connected via tubing and the other UAV with an on-board near-IR CH_4_ analyzer (ABB-pMGGA, 3.4 kg and 2.2 ppb precision at 1 Hz). In this paper, we present a small, customized rotary UAV system (6.7 kg total weight; flight time 10 min per battery pair) with the capability to directly map CH_4_ hotspots and to measure total area fluxes using on-board instrumentation only, including wind and CH_4_ concentrations at high precision and frequency (0.84 ppb and 0.1 m/s at 1 Hz) using a lightweight mid-IR CH_4_ sensor (Aeris MIRA Pico, 1.9 kg). The method can be applied generally for flux measurements of gases from point sources, large areas, whole-plant emissions, inaccessible sources in complex industrial landscapes, and for leak detection and mapping. As a case study for testing, we have mapped hotspots and measured the total CH_4_ emission from a sludge deposit at a wastewater treatment plant. Traditional methods, such as flux chambers, have proven difficult to apply to sludge deposits due to inaccessibility as they have rough surfaces with large variations in height, and disturbing surfaces during measurements would mean increasing the fluxes.

## 2 Materials and Methods

Horizontal CH_4_ mapping for hotspot and leak detection over larger surfaces (such as a landfill or a gas pipeline), especially during low horizontal wind speeds, can be made by flying in a pattern at a certain altitude above an emission area (Figure S1, panel A) while sampling geotagged CH_4_ concentrations at high frequency. The method cannot currently be used for direct emission estimates with a UAV using measured vertical wind speeds (which are often very low compared to the horizontal component), especially with the uncertainty introduced by turbulence from the propellers of a rotary UAV. During low horizontal wind speed, however, when the CH_4_ is rising close to vertically, it can be an efficient method for emission hotspot mapping over large areas or long distances.

Vertical CH_4_ mapping is, however, suitable for mass balance calculations and estimates of total emissions. It does require nonzero horizontal wind speeds, and the vertical area that needs to be mapped is dependent on the wind speed and emission footprint, as lower wind speeds and larger surface areas do require higher max altitudes to capture the full extent of emissions at the sides of the imaginary box used for mass balance calculations of fluxes from the target area (Figure S1, panel B). It can be important to map both the up and downwind sides of the box to discount CH_4_ flowing into the box from other emissions. The total CH_4_ flux from an encapsulated source can then be calculated by considering the horizontal inflow and outflow into and out of the box, with geotagged CH_4_ concentrations and horizontal wind measurements being logged on-board the UAV, flying in a vertical pattern covering both the upwind and downwind walls of the box. These two vertical patterns should preferably be flown in rapid succession (without changing UAV batteries) to minimize uncertainty from possible changes in wind conditions. To improve the accuracy of the concentration and wind maps, the patterns can be flown several times followed by calculations of average vertical maps in postprocessing for both perpendicular wind speed and CH_4_ concentrations. We used a transmitter on the UAV and a receiver connected to a laptop for real-time visualization of CH_4_ concentrations and other parameters to find the altitude limits of the plume, above where only background levels were sampled.

Our system consists of a customized DJI Matrice 210 quadcopter equipped with several on-board sensors (Table 1). In addition, the UAV itself had an internal log of GPS coordinates, altitude, air pressure, velocity, yaw, pitch, and roll that we could combine with our log using our own sensors, as a backup in case our GPS or pressure sensors did not work, and to obtain the UAV velocity and yaw that are needed when transforming measured wind velocities from UAV-relative to ground-relative coordinates. The UAV also has a visual FPV camera for navigation, an IR sensing system, and a downward vision system that in combination with GPS coordinates improves the reported positional accuracy to ±0.3 m in the horizontal and ±0.1 m in the vertical direction (up to a height of at least 10 m). This is done by tracking features on the ground with a downward camera, detecting horizontal motion of the UAV, and correcting the vertical GPS position. All our flight tracks were flown during good lighting conditions and had the improved positional accuracies turned on.

**Table 1:** UAV Instrumentation

component	weight (g)	sampling rate (Hz)
CH_4_ sensor Aeris MIRA Pico (0.1–10 000 ppm range)	1924	1
anemometer (Trisonica)	50	5
humidity sensor (Sparv)		1
GPS (Sparv and DJI)		1
pressure sensor (Sparv)		1
temperature sensor (Sparv)		1
visual camera		real-time
logger for all sensors (Sparv)	25	1
carbon fiber rod	150	

The CH_4_ sensor was an Aeris MIRA Pico (mid-IR laser gas sensor) working in the strong 3.3 μm CH_4_ absorption band, having a sensitivity of 0.84 ppb/s for CH_4_ in the range of 0.1–10 000 ppm and <0.5 ppb/s for ethane (C_2_H_6_) logging at 1 Hz. An average long-term drift of 20 ppb over the full temperature range is quoted from Aeris, a possible absolute level offset that can be calibrated in the field before flight using another instrument if very high absolute accuracy (better than the current value of ∼5 ppb; see the Supporting Information (SI)) is needed. For the total flux calculated using the mass balance method, this is not necessary, if sampling upwind and downwind concentrations, as excess concentrations are relative to the background level. Its total weight was 2.75 kg before customization, which was reduced down to 1.92 kg after removing the battery, case, and logger, instead using the UAV battery for its 15 W power consumption and a customized universal logger for all sensors. The gas sensor also measures water vapor concentrations, which are used to correct the CH_4_ concentrations to be reported as dry mole fractions.

For on-board wind measurements, we use the ultrasonic anemometer TriSonica, weighing only 50 g, mounted on top of a carbon fiber rod 40 cm above the propellers, at the same spot where air is pumped down to the CH_4_ sensor (Figure S2). It has a resolution of 0.1 m/s in the 0–10 m/s range and ±1% in the 11–30 m/s range. Data output rates are selectable in the 1–10 Hz range; we use a 5 Hz sampling rate and average this to 1 Hz for the universal logger together with the other sensors working at 1 Hz. It has a built-in compass to relate measured wind vectors in the UAV coordinate system to geographical north.

Sensor integration was made by Sparv Embedded AB, who also designed the data logger and made the necessary customizations in the form of a climate box below the UAV that protects the CH_4_ sensor, and a carbon fiber rod with a 3D-printed holder attaching it to the top of the UAV for wind measurements using an anemometer at its top (Figure S2). Through test emissions at the gas intake, located at the end of the plastic tube close to the anemometer, we have found that the delay between air sampling at the top of the rod and the sensor is 8 s, which was used in the data logs to match GPS positions and altitudes with the corresponding CH_4_ concentrations. Although turbulence is an uncertainty for concentration and wind speed measurements on a drone, this is decreased by placing the gas intake and anemometer several decimeters above the propellers and by not flying the vertical patterns very close to the sludge deposit.

The system has a total weight of about 6.7 kg (4.5 kg for the UAV with two TB50 batteries and 2.2 kg for the sensor, logger, and climate box). The maximum flight duration with this configuration is 10 min per flight and battery pair, with a total flight time of 30 min (1 800 measurements at 1 Hz) using our setup of three pairs of batteries. All sensors were powered by the UAV batteries, with a flight time that was limited by the weight of the system rather than the power usage of the detectors and logger. We also used a Vaisala weather station on the ground (1.5 m height) for comparison of wind measurements with the UAV at different altitudes (wind profiles).

**Figure 1:**
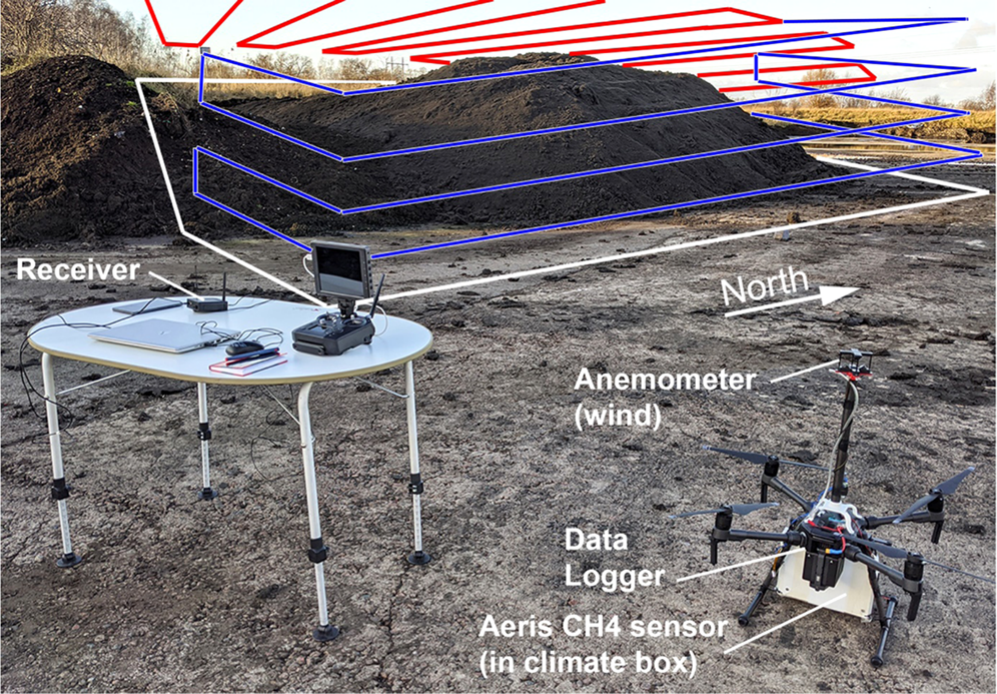
UAV system setup at the sludge deposit used for the test flights. Illustrative flight tracks are shown as red (horizontal surface above the pile for hotspot mapping) and blue curves (vertical planes for mass balance and total flux calculations). The white curve indicates the emission footprint in a no-wind situation. A receiver and laptop allowed real-time viewing of data in addition to on-board data logging.

Measurements were made for 2 days (two horizontal plane flights on 25 Nov 2019 for mapping CH_4_ hotspots and three vertical plane flights on 24th Jan 2020 for total flux calculation) at the Linköping wastewater treatment plant (Tekniska verken) in Sweden over a dewatered sludge deposit of solid material, which are known to have high CH_4_ emissions.^5^ The sludge pile (Figure 1) had a size of 30 × 25 m^2^ (750 m^2^) and a height of 3–4 m. The UAV was flown manually, at a set speed of 1 m/s for precision flight, to safely avoid obstacles (using a camera, visual inspection, and obstacle sensors) and to map CH_4_ and wind at a spatial resolution of about 1 m. The system can, however, fly at speeds up to 17 m/s to cover a larger area or to monitor a pipeline for leaks over larger distances. It is, however, important to note that very high speeds, and fast turns, will lower the accuracy of the on-board wind measurements as the uncertainty in the UAV speed would increase. Only vertical plane flights (Figure 1, blue curves) were used for flux calculations as they capture the whole plume from the emission area if the flight tracks extend to sufficiently high altitudes. Such vertical planes were flown on all four sides of the pile, also capturing the influx of air with ambient concentrations. The horizontal plane flights (red curves in Figure 1) were only part of a test for mapping hotspots in low-wind conditions (<1 m/s) on a different day as such wind speeds would mean both a relative high uncertainty in the wind data and that the UAV would have to fly a lot more flight tracks to capture the full plume from the emission.

Using the ideal gas law, we get for the mass of CH_4_ in grams within 1 cubic meter of air at measurement location (*x*,*y*)
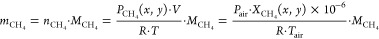
1where *X*, *P*, *T*, *R*, and *M* are the mixing ratio (ppm; parts per million by volume), pressure (Pa), temperature (K), universal gas constant (J·K^–1^·mol^–1^), and molar mass (g·mol^–1^), respectively. The location parameters *x* and *y* represent the horizontal distance along the ground and the altitude, respectively.

Using a grid size in our mapping of 1 × 1 m^2^, and denoting wind speed perpendicular to a vertical plane with *v* (m·s^–1^), the flux *F*_CH_4__ (g·m^–2^·s^–1^) was then calculated using
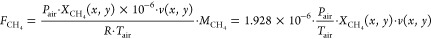
2where we used *M*_CH_4__ = 16.031 g·mol^–1^ and *R* = 8.314 J·K^–1^·mol^–1^.

The on-board anemometer gives total apparent wind speeds and wind directions relative to the direction the UAV is pointing; therefore, we first converted measured wind speeds in the UAV coordinate system (*v*_*u*_, *v*_*v*_) to ground wind speeds in the longitudinal and latitudinal directions (*v*_long_, *v*_lat_) using the UAV speed, yaw, and velocity. As a second step, the components of the wind speed perpendicular to the sides of the mass balance box were calculated, denoted as *v*(*x*,*y*).

Resampling of CH_4_ concentrations and wind speeds onto a 1 × 1 m^2^ grid on a vertical plane (similar to the method used by Allen et al.^25^) was made using partitioning into nearest grid elements, with average values being used at positions with multiple measurements. For grid elements that lacked measurements, linear interpolation was used to fill possible holes across the measurement plane. Measurements from all three flights were used to make average maps of *v*(*x*,*y*) and *X*_CH_4__(*x*,*y*) for the flux calculations.

Integrating across the extent of each vertical plane (north, east, west, and south) for the encapsulating mass balance box then gave the respective fluxes and the total emission from the sludge deposit. The flux uncertainty was calculated in several steps starting with estimating the uncertainty of each parameter (wind speed, wind direction, and CH_4_ concentrations) for each measurement point in the point cloud. This was done by calculating the standard deviation of three points adjacent in time (±1 s as measurements were made at 1 Hz) to estimate variations local in time and space. As a second step, we calculated the flux uncertainty for each vertical plane (side of the box) using the Monte Carlo method by simulating 100 parameter and flux grids, each grid based on the same actual measurements but with added values within the uncertainties of each point. As the last step, from such simulations for each vertical plane of the box, we obtained 100 total flux estimates with a total uncertainty that was given by the standard deviation.

## 3 Results and Discussion

The flights on 25 Nov 2019 were test flights of the system in low-wind conditions (<1 m/s), with flight tracks above the sludge deposit, making a CH_4_ concentration map of hotspots and to survey the range of concentrations that could be expected (Figure S1, panel A). We will mainly present results with a focus on the flights made on 24 Jan 2020 as they also included vertical flight patterns that were used to calculate the total flux using mass balance (Figure S1, panel B).

### 3.1 Methane Hotspot Mapping

**Figure 2:**
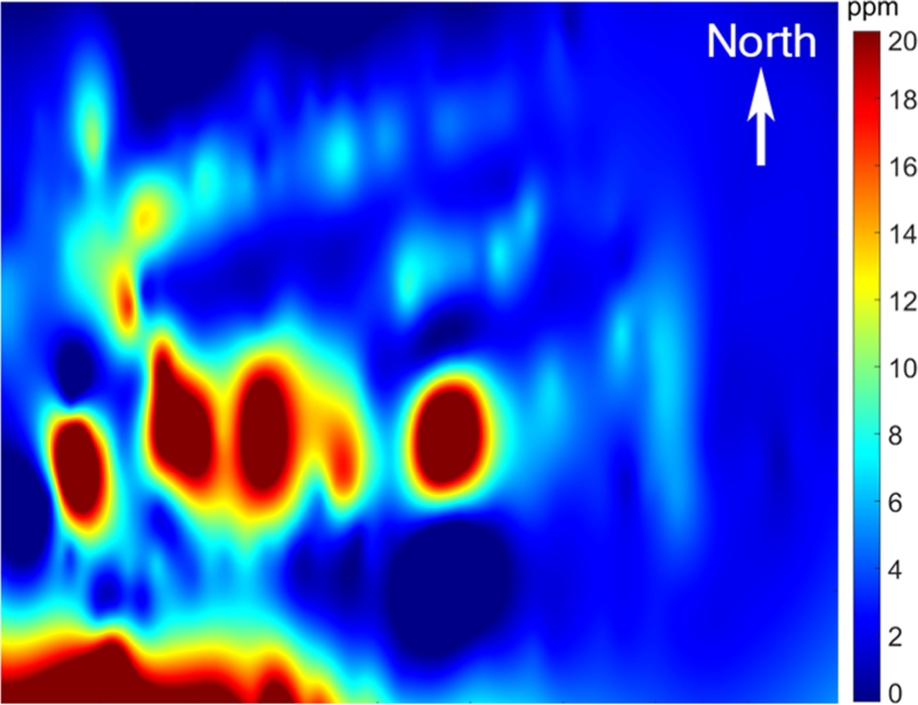
Horizontal CH_4_ concentration map for the November 2019 test flights during low wind speed, clearly showing hotspots above the sludge pile. The map has been interpolated using 1200 measurement points distributed evenly over the area with the UAV flying at an altitude of 7 m and the map having a size of 35 × 35 m^2^.

During low-wind conditions (<1 m/s), it is possible to fly horizontally above an area and make a map of CH_4_ concentrations, highlighting hotspots and revealing other emission patterns. An example of this is shown in Figure 2 for our two Nov 2019 flights (covering the area shown using red curves in Figure 1 using a point cloud of about 1200 measurements). Concentrations were in the range of 1.90–60 ppm (average value 4.43 ppm), with the highest concentrations in the SW corner of the pile, corresponding well with higher emissions from new material being recently loaded into that area, as emissions are known to decrease with time. There were also four other hotspots in the pile, clearly visible in Figure 2, that were not identified as high-emission areas from visual inspection beforehand. One reason for such structures, in areas with older material, could be wind-sheltered surfaces with higher residence times of the gas, building up higher concentrations over time. During these test flights, however, the wind speed was negligible, indicating higher emissions (at least by a factor of 10) that could have been caused by structural differences such as cracks in the surface material or volumes of more porous material.

Two of the Jan 2020 flights also had flight tracks above the pile (altitude 7 m) in a horizontal plane, although more focus was given on the vertical planes for mass balance calculations as the wind speeds were much higher that day. From these measurements, two horizontal CH_4_ concentration maps were made (Figure S3), both showing very high concentrations at the downwind (east) side of the pile. Concentrations were in the range of 1.90–53 ppm (average value 5.41 ppm). Horizontal CH_4_ concentration maps can be used to estimate total fluxes from an area by using inverse plume modeling, identifying sources, and comparing modeled and measured CH_4_ concentrations in an optimization procedure.^20^ In our project, we used such maps to pinpoint hotspots, investigate spatial emission variability, and to test our UAV system. Total fluxes were, however, calculated from mass balance, using only vertical plane CH_4_ mapping around the entire emission area (see below), which requires less assumptions regarding atmospheric stability and emissions from individual hotspot sources.

### 3.2 GPS Tracks

The Jan 2020 flights consisted of two horizontal flight patterns (flights 2 and 3) and for the mass balance calculation of three vertical patterns (flights 1–3) to give better average CH_4_ and wind maps for the flux calculations (Figure S4). The GPS-only positional accuracy is ±1.5 m; however, also using the Matrice 210 downward vision system (as we flew below 10 m) had an improved accuracy of ±0.3 m.

### 3.3 Pressure and Altitude

As the altitude obtained from GPS sensors typically has an uncertainty of several meters, we instead used pressure sensors (Sparv and DJI) to calculate relative altitude from the launch position. The pressure curves from the DJI’s built-in sensor and our external sensor (Figure S5) served one additional purpose besides calculating flight altitude; they were used to synchronize the clocks of the two loggers to allow combining data from both logs as we needed the velocity and yaw of the UAV in our flux calculations. For altitudes up to 10 m, which was the case for all our mass balance calculations, we used the altitudes obtained with the downward vision system turned on, having an improved accuracy of ±0.1 m.

### 3.4 Wind Measurements

**Figure 3:**
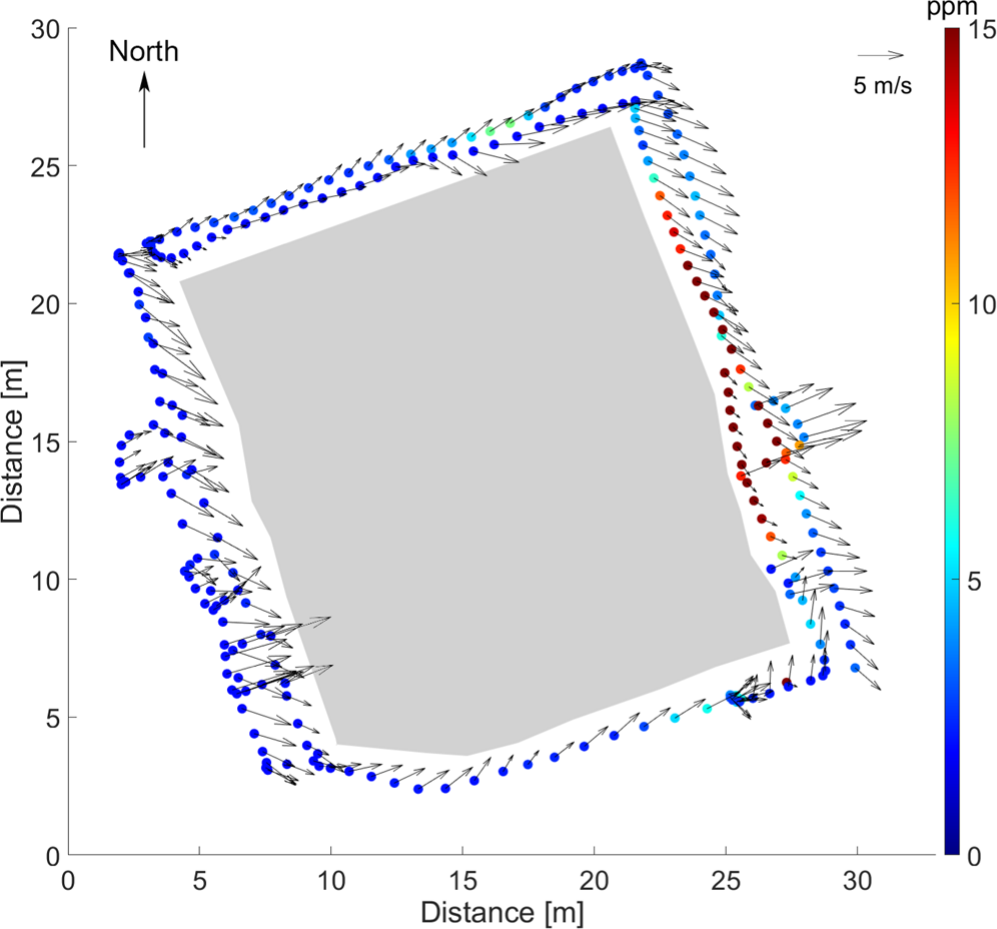
Example of CH_4_ concentrations and wind vectors for part of flight 3 around the edges of the pile (several altitudes combined). The wind vectors have been corrected for the motion and yaw of the UAV. The lengths of the arrows correlate with wind speeds, with a 5 m/s arrow drawn in the top-right corner as a scale reference. The sludge deposit is outlined by the gray area.

Wind speeds were very different between the on-ground weather station at a 1.5 m height and the UAV’s anemometer when flying higher up (Figure S6), showing the need to have an on-board weather station for accurate flux calculations. As a test, we flew the UAV up to a 23 m altitude, where the wind speed was as high as 10 m/s, at the same time only 3 m/s as measured by the on-ground weather station. At low altitudes, the on-board and ground weather station average wind speeds agreed (Figure S6), although the on-board anemometer has a much higher sampling frequency, capturing fast variations in wind. With increased altitude, the deviation between the on-board and ground station wind speeds increased, showing the importance of mapping both wind and gas concentrations on the UAV. The motion- and compass-corrected wind directions (Figure 3) agree with the average wind direction of the ground weather station but also shows important local variations due to small-scale air motion. Bailey et al.^29^ have shown that placing the TriSonica anemometer 0.38 m above the main body of their quadrotor UAV (similar to our 0.40 m mast height) was optimal for wind measurements as this was the smallest mast height where running the propellers at full speed did not affect anemometer readings. They also showed that wind data were reliable except for vertical profiles, where high ascend or descend speeds cause the vertical air speed to partly be measured as horizontal wind speed. The flight paths in our study involved horizontal flight tracks with vertical flight tracks only for a short time when changing the altitude by about 1 meter at a time with slow vertical speed, which should have a negligible effect on our wind data.

Combining the CH_4_ concentrations and wind vectors (Figure 3), it is clear that the outflow from the area is mostly on the east (right) side and that lower wind speeds are connected to higher concentrations as expected due to longer residence time. Combining the measurements from all three flights, measurement points located outside the edges of the pile could then be extracted and indexed using Matlab and processed further.

### 3.5 Mass Balance and Total CH_4_ Flux

For the vertical plane flight tracks, the UAV was flown at a maximum altitude of 7 m to increase the signal-to-noise ratio of the mapping (higher concentrations). During flight 2, we also sampled a vertical profile in the altitude range of 0–23 m starting on the ground at the downwind side of the sludge deposit to map the vertical extension of the plume from the deposit (Figure S7). It is clear that there was a large variation in CH_4_ concentrations up to a height of about 7–8 m, with background concentrations at higher altitudes (similar to the concentrations on the upwind side, below 2 ppm). This justifies the selected max. altitude used in our vertical cross sections, indicating that the cross sections capture the total emission from the enclosed area.

**Figure 4:**
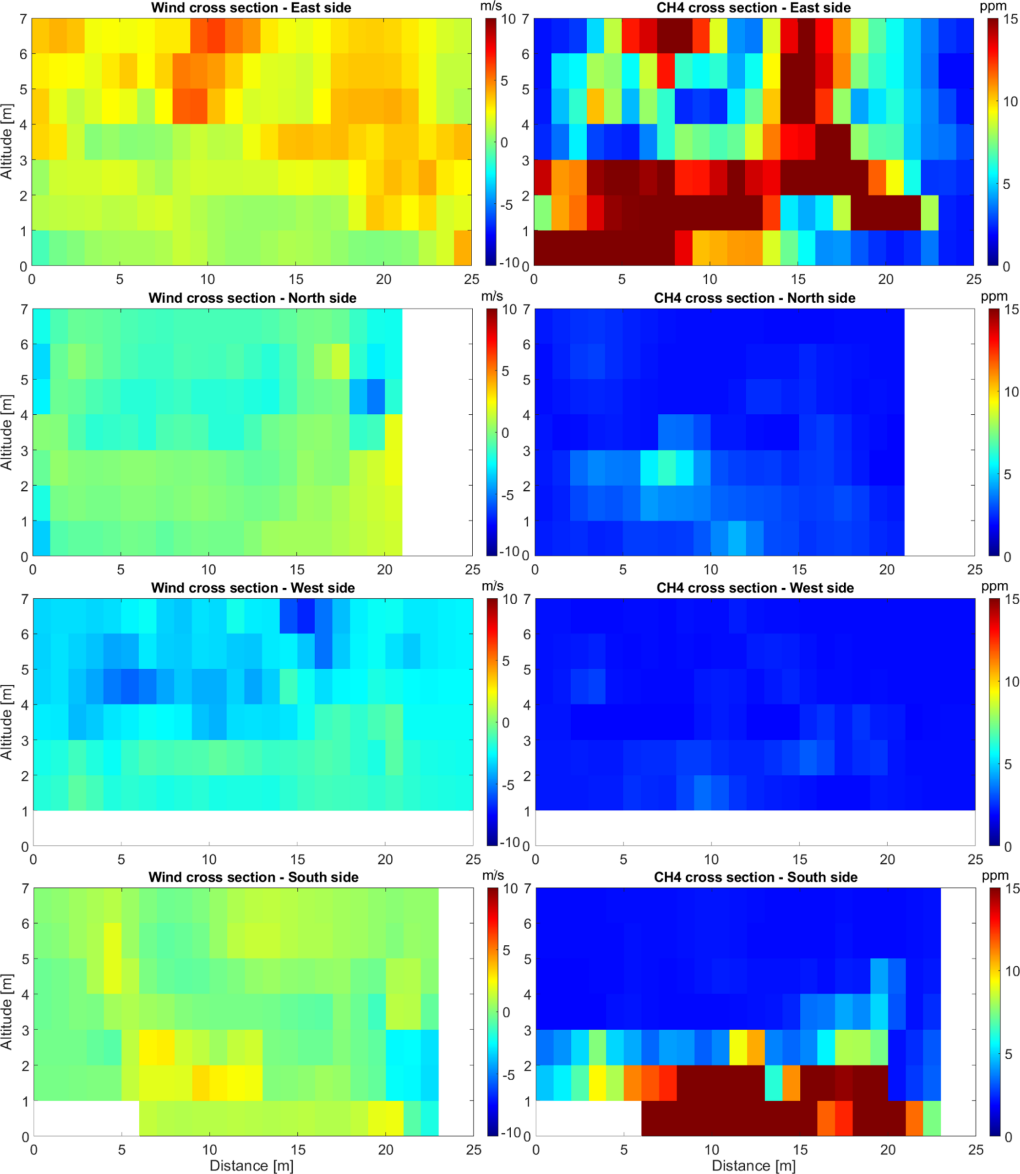
Cross sections of the average wind speed (perpendicular to the vertical surface) and CH_4_ for the four sides of the sludge deposit below 7 m above ground level, used for mass balance calculations of total CH_4_ emissions. Positive and negative wind speeds indicate that there is an outflow or inflow through that vertical surface, respectively. The lowest meter is missing for the west side and parts of the south side as the ground was elevated by 1–1.5 m along these sides. Tracks at higher elevation showed minor deviation from background air at all sides and are therefore not shown (see Figure S7).

Measurements in the point cloud were divided into five categories: east, north, west, and south wall, and above the pile (see Figure S8 for plots of CH_4_ concentrations in the four vertical planes). For each wall of the mass balance–volume, we made a 1 × 1 m^2^ resolution map of CH_4_ concentration and wind speed perpendicular to the vertical surface by partitioning each measurement point into the four nearest grid elements (for *v*(*x*,*y*) and *X*_CH_4__(*x*,*y*)) with relative contributions according to their coordinates using bilinear interpolation (Figure 4).

Integrating across the extent of each wall, we calculated the ingoing and outgoing fluxes and the total emission from the sludge deposit (Table 2), including uncertainties in the estimates (see the Materials and Methods section for details). These fluxes are based on absolute CH_4_ concentrations (including the background level, as can be seen by the inflows of CH_4_ at the upwind north and west sides). The total emission from the sludge deposit was calculated to be 178.4 ± 8.1 kg CH_4_ d^–1^ based on the three flights on 24 January 2020.

**Table 2:** CH_4_ Fluxes from the Different Sides of the Sludge Deposit and Total Flux from Mass Balance^a^

component	flux (kg CH_4_ d^–1^)
east side	+196.2 ± 6.0
north side	–11.5 ± 0.7
west side	–50.3 ± 0.9
south side	+44.0 ± 5.3
total CH_4_ flux	178.4 ± 8.1

a Plus and minus signs indicate the net fluxes out from and into the area, respectively.

The sensor long-term drift is negligible during the flight time of the UAV (10 min) but has been seen in lab conditions to be up to 20 ppb after a very fast increase in air temperature of 5 °C, giving transients lasting 10–20 min. Meteorological changes are, however, rarely this fast; we therefore expect drifts of less than 10 ppb in an hour for typical field environments. Such variations are therefore also negligible for longer flight times (using several battery pairs), especially relative to the measured variations in actual CH_4_ concentrations of many ppm. After stabilization at a site, the sensor has a precision of 0.84 ppb/s at 1 Hz and an accuracy better than 5 ppb (which can be lowered to close to 0 if the instrument is recalibrated in the field prior to a flight), as shown in lab experiments with durations up to 140 h. An in-depth description of materials and the sensor performance is given in the SI.

In a prior study at the same wastewater plant (unpublished) lasting a year and targeting all of the treatment steps of the wastewater treatment plant, we measured CH_4_ concentrations above young (<1 month) sludge deposits (close to their edges as this could be reached) on seven occasions using a Los Gatos Ultraportable Greenhouse Gas Analyzer (UGGA) and found concentrations in the range of 2.0–63 ppm; this is in agreement with the concentrations found with the Aeris instrument on the UAV. In that study, we also made ground-based hyperspectral remote sensing measurements^13,14^ of total fluxes from sludge deposits, which were in the range of 62–220 kg CH_4_ d^–1^ for ages 0–30 days, also in agreement with the total flux from the present UAV method study.

In the present study, we measured four geographical vertical planes (east, north, west, south) for the mass balance calculations, as well as horizontal flight tracks above the sludge deposit for CH_4_ hotspot mapping. A future method improvement for total flux calculations would be to map only the downwind vertical plane (or possibly two if the geographical planes are used due to nearby obstacles such as buildings) if the upwind (background) concentrations can be assumed to be sampled at the highest altitudes in the downwind sampling. This would allow 30 min of flight time (three pairs of batteries) with roughly 1800 measurement points available for the downwind side and three times longer flight time if using a more powerful UAV such as the DJI Matrice 300 RTK. Flying at higher speeds in an environment with fewer obstacles and measuring a more extended emission area such as a landfill, this would make flux estimates possible for plumes extending hundreds of meters horizontally and up to 120 m altitude (the current maximum allowed flight height in Sweden).

This study confirms the potential of drone-based CH_4_ concentration mapping and flux measurements to contribute a valuable supplement to established greenhouse gas measurement techniques. With further optimization of drone size versus sensor payload, flight times and areal coverage can be increased. The capability to effectively integrate all sensors needed to derive fluxes on the drone opens up for very effective flux assessment at widely different scales, which is of high value to bridge the scaling gap between m^2^ flux chamber studies and ha to km^2^-sized micrometeorological or remote sensing-based approaches. Further tests of drone measurements in more types of environments would be helpful to reveal the full capacity as well as the pros and cons for this novel promising approach.

## 4 Conclusions

We demonstrated our newly developed drone-based CH_4_ system and method by mapping high-emission hotspots and calculating the total emission from a sludge deposit at a wastewater treatment plant (178.4 ± 8.1 kg CH_4_ d^–1^ from three 10 min flights). All measurements were made on-board the drone, without the need for any ground-based instrumentation, logging both CH_4_ concentrations and wind every second (0.84 ppb and 0.1 m/s precision). The method is general, with applications in many anthropogenic and natural environments including emissions from extended areas that are difficult to measure using traditional methods such as flux chambers, searching for leaks from gas pipelines, and mapping and measuring of inaccessible emissions in complex industrial landscapes.

## Supporting Information Available

Flight strategies and methods for source visualization and flux measurements (Figure S1); drone system with an on-board anemometer (Figure S2); horizontal plane CH4 concentration maps (Figure S3); flight tracks for all of the measurements (Figure S4); comparison between our integrated pressure sensor and the DJI built-in pressure sensor (Figure S5); comparison between UAV and ground weather station wind speeds (Figure S6); height profile of wind and CH_4_ concentration (Figure S7); and cross sections of the flight tracks below 7 m (Figure S8) (PDF)
