# Supplementary material for: Sensitive Drone Mapping of Methane Emissions without the Need for Supplementary Ground-Based Measurements
Source: ACS Earth Space Chem. 2021 Jul 28;5(10):2668–76. doi: 10.1021/acsearthspacechem.1c00106 (PMC8543601; doi:10.1021/acsearthspacechem.1c00106)
Supplement: Supplementary file 1 — sp1c00106_si_001.pdf [file sp1c00106_si_001.pdf]

Supporting Information for “Sensitive drone mapping of methane emissions without the need for supplementary ground-based measurements”

Magnus **Gålfalk**[1]*, Sören **Nilsson-Påledal**[2], David **Bastviken**[1]
(family names written in bold)
*Corresponding author
[1]Department of Thematic Studies – Environmental Change, Linköping University, 581 83 Linköping, Sweden.
e-mails: magnus.galfalk@liu.se, david.bastviken@liu.se
[2]Tekniska verken i Linköping AB, Box 1500, 581 15 Linköping, Sweden
e-mail: Soren.Nilsson-Paledal@tekniskaverken.se

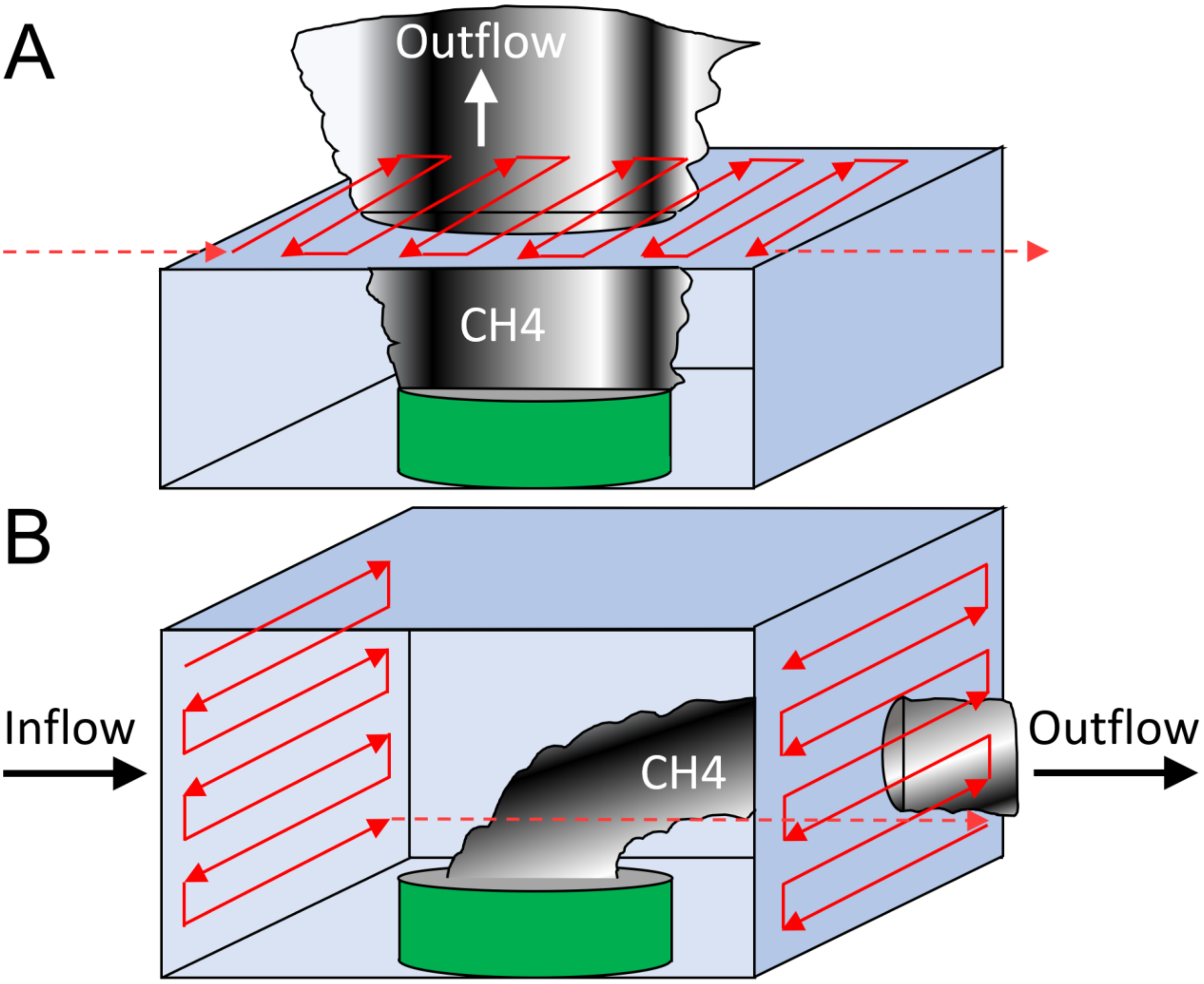

Figure S1. Flight strategies and methods for source visualization and flux measurements of CH_4 using a rotary UAV and mass balance calculations. Horizontal surface concentration map for finding sources across large areas, especially during low wind speeds (**A**) and vertical concentration maps for calculating total CH_4 emissions at higher wind speeds (**B**). Flight paths are shown using red arrows in both panels.

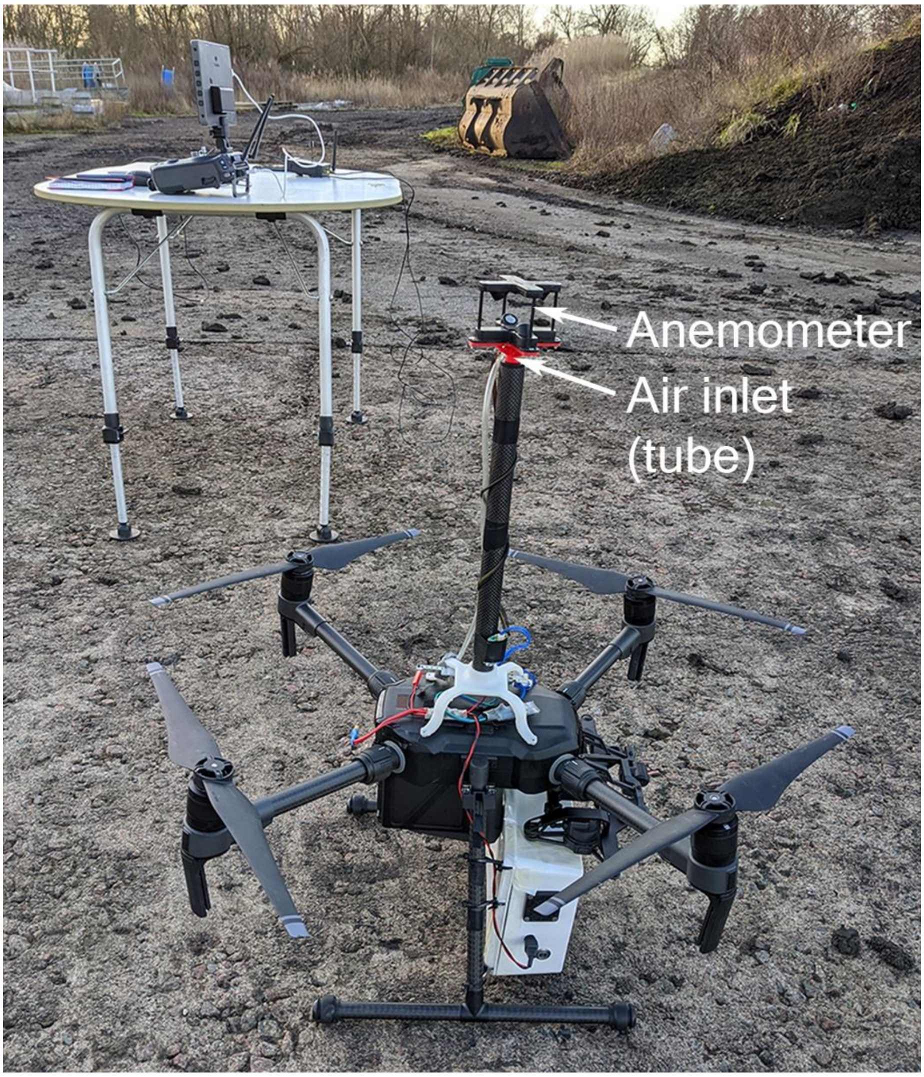

Figure S2. Drone system with on-board anemometer on top of a carbon fiber rod and an optical CH_4 instrument inside a climate box below the drone with air being pumped down in a tube so that wind and CH_4 concentrations are measured at the same location. The anemometer and air inlet (marked with arrows) are located 40 cm above the propellers.

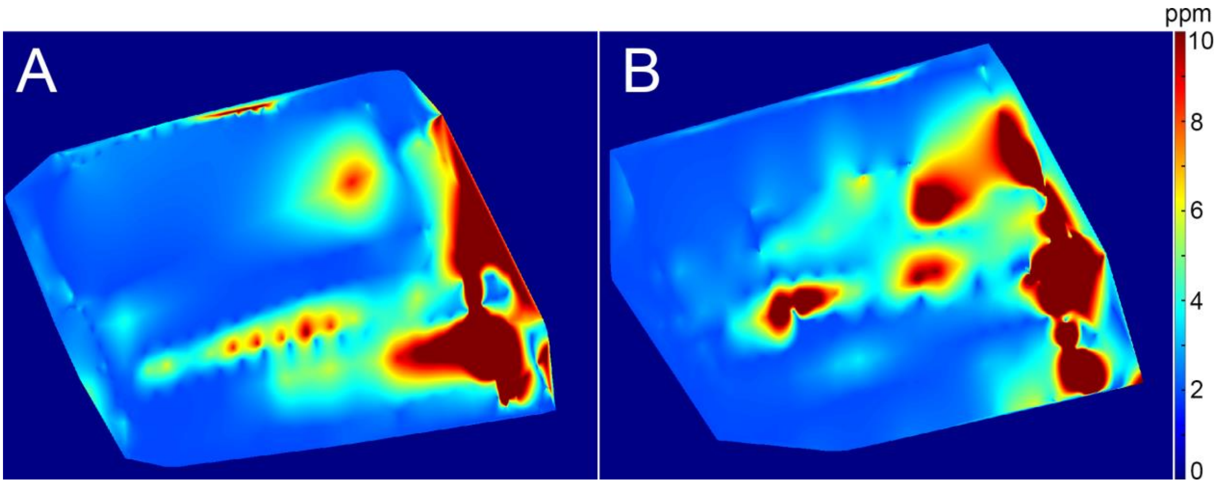

Figure S3. Horizontal plane CH_4 concentration maps from flights 2 (**A**) and 3 (**B**) on 24 Jan 2020 at a height of 7 meters above ground. Both show a similar pattern with high concentrations on the downwind side of the area (east side). North is up in these maps.

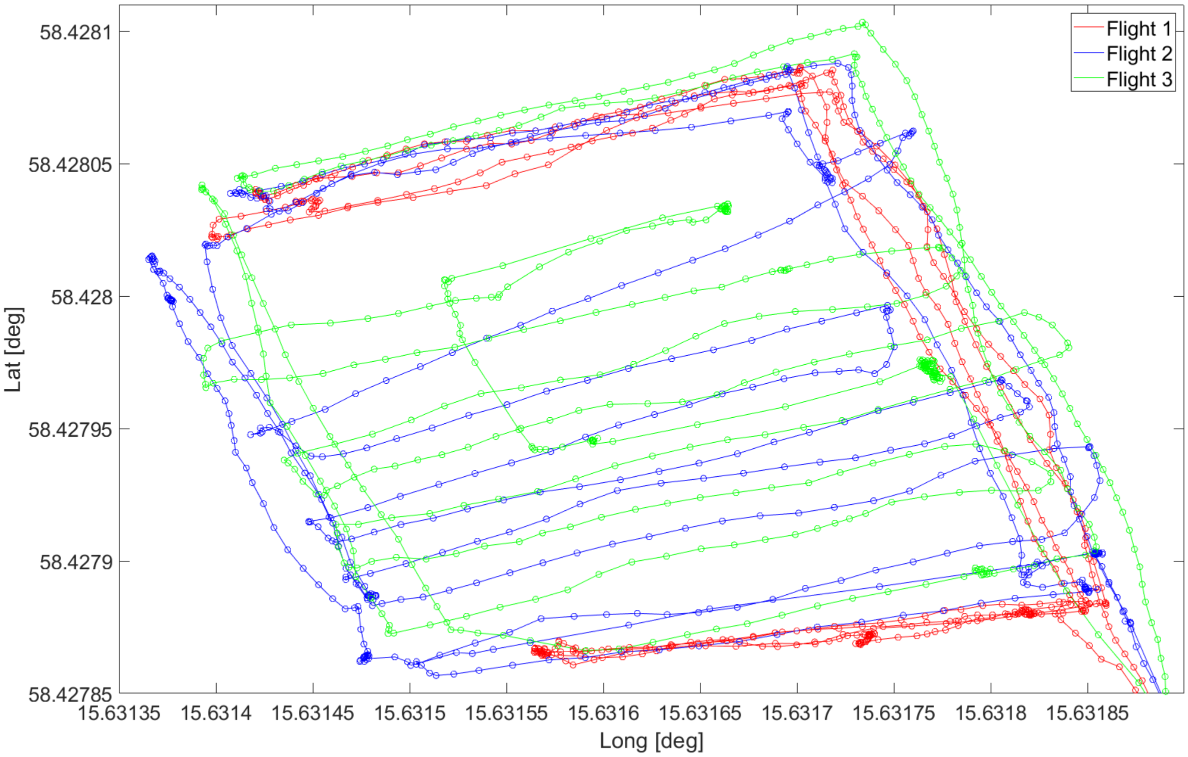

Figure S4. Flight tracks for all the measurements made on 24th of Jan 2020. Flight 1 consisted of vertical planes to the side of the pile, while flights 2 - 3 also included a horizontal plane above the pile. In total there were 1 625 measurements made during these flights, around and above the pile.

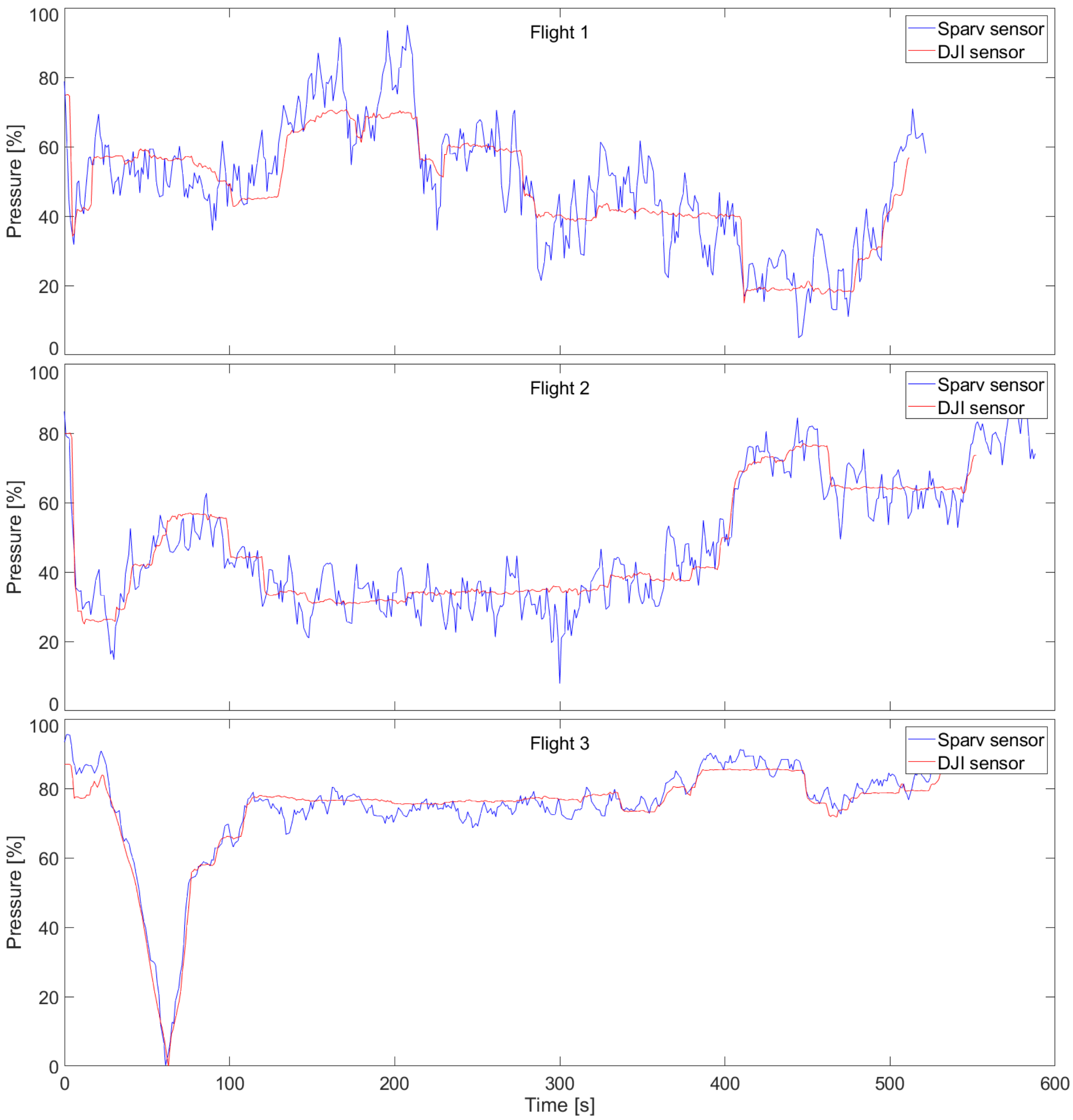

Figure S5. A comparison between our integrated pressure sensor (Sparv) and the DJI built-in pressure sensor (extracted from flight data). As different units were used they have been normalized to 0 – 100% to be used for synchronization of time for the Sparv and DJI loggers.

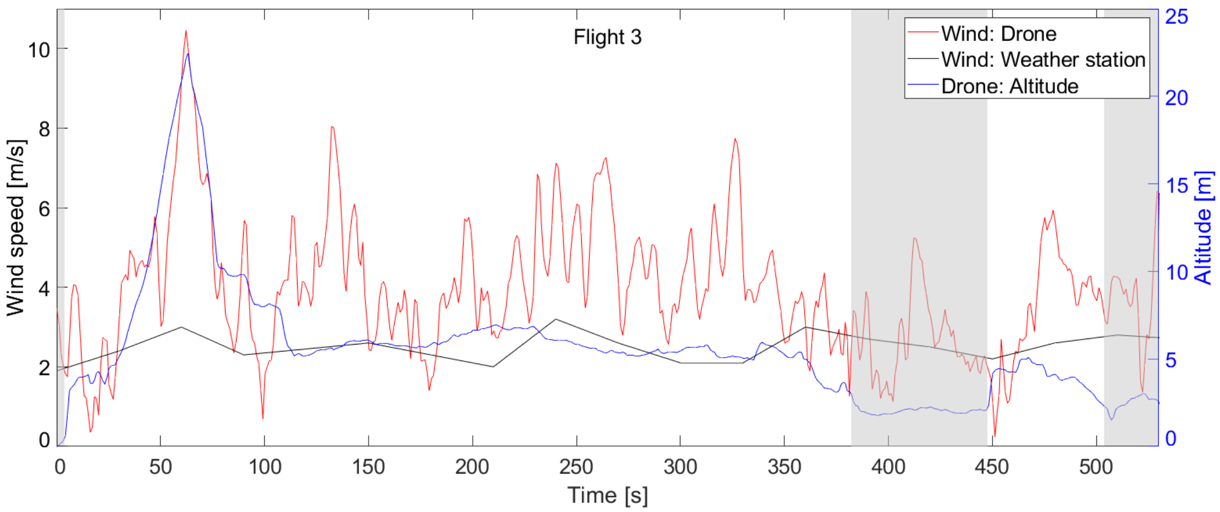

Figure S6. A comparison between UAV and ground weather station wind speeds for flight 3 shows that there is a much higher variability in the UAV measurements, especially at higher altitudes, highlighting the need to have on-board wind measurements. At low altitudes (gray shaded areas) the UAV and ground anemometers are in rough agreement (taking into account the much higher frequency of the UAV measurements). The ground weather station measured winds at an altitude of 1.5 m.

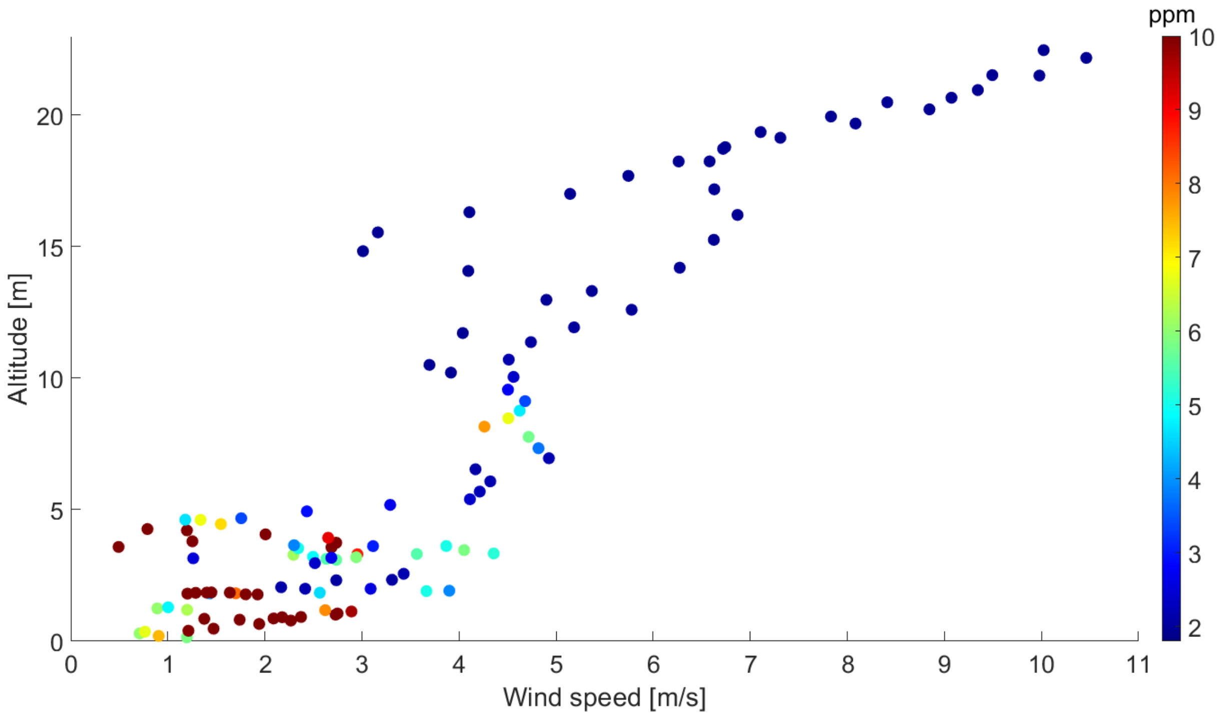

Figure S7. Height profile of wind and CH_4 concentration obtained from an ascent and descent between 0 and 23 meters. There is a large variation in CH_4 concentrations up to about 8 m and ambient concentrations < 2 ppm above this. Wind increased with altitude up to > 10 m/s at 23 m altitude which is roughly the maximum wind speed for flying the UAV safely.

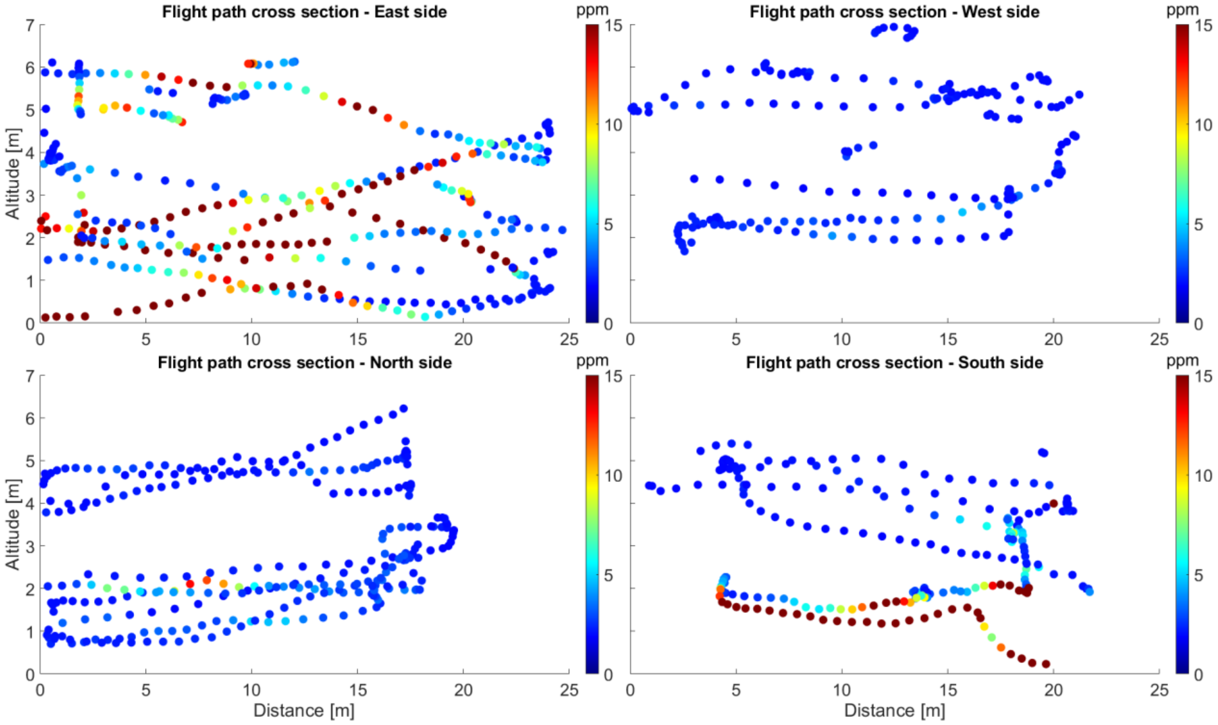

Figure S8. Cross sections of the flight tracks below 7 m for the four sides of the sludge deposit. Measurements are colored according to CH_4 concentration. Tracks at higher elevation showed minor deviation from background air at all sides and are therefore not shown.

1. In-depth description of materials

The gas sensor was a MIRA Pico (https://aerissensors.com/mira-pico-mobile-lds) made by Aeris Technologies, using mid-IR laser absorption spectroscopy, allowing simultaneous measurements of methane (CH_4), ethane (C_2H_6) and water vapor (H_2O). We 3D-printed a plastic casing for the sensor and removed the logger and batteries that came with the sensor, with the total weight for the sensor and casing being 1.92 kg. A TriSonica anemomenter (https://anemoment.com) (50 g) was placed on the top of a carbon fiber rod (150 g) and connected to a logger together with meteorological sensors (25 g) for a total weight of 2.2 kg. The UAV was a DJI Matrice 210 with two TB50 batteries (4.5 kg) giving a total takeoff weight of about 6.7 kg. Integration of sensors and logger system was done by Sparv Embedded (http://sparvembedded.com).

The gas sensor has a 15 W power consumption and was connected to the UAV batteries. Measurements were made at 1 Hz and logged, together with data from the meteorological sensors, using the Sparv logger. Using a small (60 cc volume) measurement cell minimizes the signal response time and allows measurements of fast-changing air concentrations. A pump is used to feed the measurement cell with air from the intake (the end of a plastic tube) located on top of the carbon fiber rod close to the anemometer (40 cm above the propellers). We measured a delay of 8 seconds between air intake and measurement, as seen from test emissions at the top of the rod. The Aeris gas sensor is based on the same well-established laser spectroscopy technique as for instance the Los Gatos Research instruments but having a lower weight and portable size. It also have low requirements for maintenance as a lower reflectivity can be used for its mirrors by working in the mid-IR (where absorption lines are stronger) instead of at near-IR wavelengths. Extensive lab tests of the gas sensor made by Aeris (personal communication) has shown a precision of 0.84 ppb/s after temperature stabilization. Several tests have been made to determine its long-term drift, with air temperature by far being the environmental parameter affecting this drift the most. At const temperature the drift is < 1 ppb (tested using a compressed air tank in a laboratory environment over a 9-hour period). As a test of a worst-case scenario, after a very quick (almost instantaneous) temperature change of 5°C tests show a transient period lasting 10-20 minutes (maximum 20 ppb offset in concentrations right after the temperature change in raw non-averaged data, followed by returning to the level prior to the temperature change). A long-term test having a duration of 160 hours, with slower temperature variations of 5°C shows a long-term peak-to-peak drift of 10-20 ppb. From these tests, at shorter time-scales such as the flight time of our UAV (10 minutes) and for an hour of consecutive flights, in typical field conditions with slow-changing air temperature, we expect an accuracy better than 5 ppb. Such a small drift in concentrations is negligible compared with the measured variations in CH_4 being in the range 1.90 – 63 ppm (also in agreement with separate test measurements made from the ground using a Los-Gatos UGGA instrument).
